# Trends in Deaths Attributable to Smoking in China, Japan, United Kingdom, and United States From 1990 to 2019

**DOI:** 10.3389/ijph.2022.1605147

**Published:** 2022-09-15

**Authors:** Haoyu Wen, Cong Xie, Fang Shi, Yan Liu, Xiaoxue Liu, Chuanhua Yu

**Affiliations:** ^1^ Department of Epidemiology and Biostatistics, School of Public Health, Wuhan University, Wuhan, China; ^2^ Hubei Provincial Center for Diseases Control and Prevention, Wuhan, China

**Keywords:** smoking, trends, deaths, decompose, age-period-cohort model

## Abstract

**Objectives:** This study aimed to estimate the long-term trends of deaths attributable smoking in China, Japan, the United Kingdom (UK) and the United States (US).

**Methods:** Using 2000–2019 death data from Global Burden of Disease (GBD) 2019, we estimated age-period-cohort effects on smoking attributable mortality, and decomposed of differences in smoking-attributable deaths in 1990 and 2019 into demographic factors.

**Results:** From 1990 to 2019, smoking-attributable deaths increased in China, which was due to population growth and demographic aging. From 1990 to 2019, both age-standardized smoking attributable mortality rates trended downward across countries. Among four countries, age rate ratios (RRs) for smoking-attributable mortality increased with age, while period and cohort RRs decreased with year.

**Conclusion:** The age-standardized mortality rates, period effects and cohort effects of smoking attributable mortality in China, Japan, UK, and US have been declining in both sexes from 1990 to 2019, which suggests that smoke-free policies, help to quit tobacco use, improved health education, more accessible healthcare service, and increased taxes have been effective. Additionally, increased smoking attributable deaths in elderly should got more attention.

## Introduction

In 2019, there were currently 1.14 billion current smokers worldwide, of which 341 million (approximately 30%) lived in China [1]. Smoking has long served as the single most preventable cause of incidence and mortality [2], and long-running cohort studies have shown that up to two-thirds of long-term smokers will eventually die from smoking-related diseases [3–5]. According to the global burden of disease (GBD) study, in 2019, smoking was the second leading risk factor for female (with 1,510.12 thousand deaths worldwide) and the first risk factor for male (with 6,183.25 thousand deaths worldwide) [6]. China has always been regarded as the country with the highest smoking attributable disease burden worldwide (first in smoking-attributable deaths in 2019, 2,418.67 thousand), and United States (US) (third, 527.74 thousand), Japan (sixth, 199.40 thousand), United Kingdom (UK) (10th, 119.78 thousand) these countries are also hold huge health losses attributable to smoking.

Smoking are leading risk factors for non-communicable diseases (NCDs), with approximately one in five NCD-related deaths being attributable to smoking in 2019 [6]. The United Nations Sustainable Development Goals (SDGs) propose to “reduce premature mortality from non-communicable diseases by one third through prevention and treatment” and “strengthen implementation of the World Health Organization (WHO) Framework Convention on Tobacco Control in all countries, as appropriate” [7]. Tobacco control has been identified as a critical and necessary part of achieving these goals [8–10], and an in-depth analysis of smoking attributable deaths is urgently needed.

China, a developing country, has the highest number of deaths attributable to smoking worldwide. It is an important topic to explore the reasons behind the trend of smoking attributable deaths in China. US and UK are representative developed countries, and Japan is a developed country with a similar culture and geographical location to China. Exploring the trends of smoking attributable deaths in Japan, UK and US could facilitate more accurate projections of its future development in China, and targeted prevention and control strategies. However, none of the existing studies on smoking attributable deaths had explored differences between age-groups in China, Japan, UK, US, and all of them lacked changes in smoking attributable deaths in different cohort as generations change [11–13]. Furthermore, there has been no comprehensive analysis of changes in smoking attributable deaths. Therefore, we aimed we aimed to focus on the comparison of smoking attributable mortality in China, Japan, UK, and US, using Das Gupta’s method to decompose the difference in smoking attributable deaths between 1990 and 2019, and explore the age, period, and cohort effects independently by sex under the age–period–cohort (APC) framework. The findings of this study could inform the allocation of resources to prevent deaths from smoking-related diseases.

## Methods

### Data Sources

The data used in this study were obtained from global burden of disease (GBD) 2019. GBD 2019 estimated attributable mortality for 87 risk factors in 204 countries and territories at the global level [6]. GBD 2019 estimates smoking attributable deaths as the total deaths multiplied by the population attributable fraction (PAF) for pairs of smoking related risk outcomes by age, sex, and year. Detailed methods for the estimation of the death from disease in GBD 2019 are presented in published studies [14].

In GBD2019, smoking cases are defined as current smoking of any tobacco product and former smoking of any tobacco product. Current smokers were defined as individuals who currently use any smoked tobacco product on a daily or occasional basis, and former smokers were defined as individuals who had quit smoking for at least 6 months. Raw data on smoking exposure come from a variety of sources, including nationally representative cross-sectional household surveys, demographic health surveys, multiple indicator cluster surveys, and living standards measurement surveys, self-reported tobacco exposure from cross-sectional surveys, the global adult tobacco survey and the World WHO STEPS surveys and published studies. Based on the above data of smoking exposure, spatiotemporal Gaussian process regression and DisMod-MR 2.1, a Bayesian meta-regression method, were used to estimate consistent current and past smoking prevalence.

In this study, the exposure level associated with minimum risk, known as the theoretical minimum risk exposure level, for smoking was 0 cigarettes per smoker per day and pack-years. Population attributable fraction (PAF) is defined as the proportion of related diseases or deaths in the population would reduce if the exposure of a certain risk factor was reduced to the theoretical minimum exposure level in a certain population [15]. Mortality attributable to smoking was estimated based on defining PAF through combining the distribution of exposure to smoking with exposure-risk estimates at each level of exposure:

$$PAF=\frac{\overset{n}{\underset{i}{\sum }}p_{i}\left({RR}_{i}-1\right)}{\overset{n}{\underset{i}{\sum }}p_{i}\left({RR}_{i}-1\right)+1}$$

where $p_{i}$ is the percentage of the population exposed to level *i* of smoking, *n* is the total number of exposure level, and ${RR}_{i}$ is the relative risk at exposure level *i*, estimated from dose-response risk curves for multiple risk-outcome pairs, the specific methods are outlined in a previous study.

Smoking attributable deaths were calculated by multiplying the PAF of smoking and the number of the deaths [6]. All estimated numbers and rates of the smoking attributable deaths in this study were reported with the 95% uncertainty interval (UI). The 95% UI was estimated by a posteriori simulation of 1,000 samples, whose upper and lower bounds were derived based on the 2.5th and 97.5th percentiles of the uncertainty distribution. The age-standardized smoking attributable mortality rate was calculated by the world standard population 2019. Therefore, we obtained the data of population and smoking-attributable death for China, Japan, UK, and US from GBD 2019. Ethical approval was not needed for this study because there was no direct involvement of human subjects.

### Methods

#### Das Gupta’s Method

Since Das Gupta’s method was published in 1993 [16], this method has been used to perform death decomposition in numerous disease burden and epidemiological studies [17, 18]. Changes in smoking attributable death were decomposed into three drivers: population growth, changes in population age structures, and changes in age-specific and cause-specific mortality.

First, we express the smoking attributable deaths as the product of three factors:

$${Death}_{y}=\underset{a}{\sum }{pop\;size}_{y}∙\frac{{pop\;age}_{a,y}}{{pop\;size}_{y}}∙\frac{{death}_{a,y}}{{pop\;age}_{a,y}}$$

where a is the age group, and y is year.

For simplicity, let *R* be the smoking attributable deaths by age; *A* be the size of the population (the first term on the right-hand side); *B* be the age structure of the population (second term); *C* be the age-specific mortality rate for each age group (third term).

Then, we can simplify the identity for the two time periods of interest into:

$$R_{1990}=A_{1990}B_{1990}C_{1990}$$

$$R_{2019}=A_{2019}B_{2019}C_{2019}$$

To estimate the additive contribution of *A*, Das Gupta’s method assumes that *B* and *C* remain the same over time by applying a standardized rate of *B* and *C*. The additive contribution of population size, *A*, on the change in smoking attributable deaths between 1990 and 2019 is then calculated as:

$$\left(A_{2019}-A_{1990}\right)\times \left[\frac{B_{2019}C_{2019}+B_{1990}C_{1990}}{3}+\frac{B_{2019}C_{1990}+B_{1990}C_{2019}}{6}\right]$$

where the latter term in square brackets are the *BC*-standardized rates for the two time periods. We follow the same step to estimate the contributions of *B* and *C*.

#### APC Model

The aim of the APC analysis was to assess the age-, period-, and cohort-effect on health outcomes (such as morbidity and mortality) [19–21]. Age effects represent the accumulation of exposures and age-related developmental changes, period effects represent changes in medical and diagnostic techniques, disease classification, culture, and economics specific to a given period, cohort effects represent early life effects on socioeconomic, behavioral, and environmental exposures. Using APC analysis, we mainly focused on the following estimable functions [1]. The net drift, the overall log-linear trend by calendar period and birth cohort, which represents the annual percentage change of the expected age-adjusted rates over time [2]. The local drifts, the log-linear trend by calendar period and birth cohort for each age group, which represents the annual percentage change of the expected age-specific rates over time [3]. The longitudinal age rate ratios (RRs), which represents the age relative risk versus a reference age group in a reference cohort adjusted for period deviations [4]. The period (or cohort) RRs, which represent the cohort (or period) relative risk adjusted for age and nonlinear period (or cohort) effects in a cohort (or period) versus the reference one.

To conduct APC analysis, the mortality and population data were arranged for consecutive 5-year periods from 1990 to 2019 and successive 5-year age intervals from 30 to 34 to 75–79 years. Because the smoking attributable death in those aged <30 years were rare in GBD 2019, and individuals >80 years were recorded as only one group in GBD database, they were not considered in APC model. We obtained the estimable parameters through the APC Web Tool [22]. In APC analyses, the central age group, period, and birth cohort were defined as reference, respectively, in this study the age group 55–60, the period 2000–2005, and the cohort 1950. Wald Chi-Square tests were adopted for the significance of the estimable functions. All statistical tests were two-sided, and *p* < 0.05 was considered statistically significant.

## Results

### Trends of Smoking Attributable Deaths

Supplementary Figure S1 shows smoking attributable deaths in China, Japan, UK, and US in 1990 and 2019, with smoking attributable deaths in China significantly higher than in other countries. In 2019, there were 2093.12 thousand male and 325.54 thousand female died from smoking in China. Between 1990 and 2019, deaths attributable to smoking increased significantly in China, slightly increased in Japanese male and US female, and decreased in other populations. The percentage change in deaths attributable to smoking between 1990 and 2019 ranged from −36.89% in UK male to 58.90% in Chinese male. As for the deaths attributed to smoking by age group (see in Supplementary Figure S2), compared with 1990, China, Japan, and US are closer in the younger age group, while those in the 80+ age group have greatly increased in 2019. Smoking attributable deaths decreased in younger age groups and remained stable in older age groups in the UK from 1990 to 2019.

**FIGURE 1:**
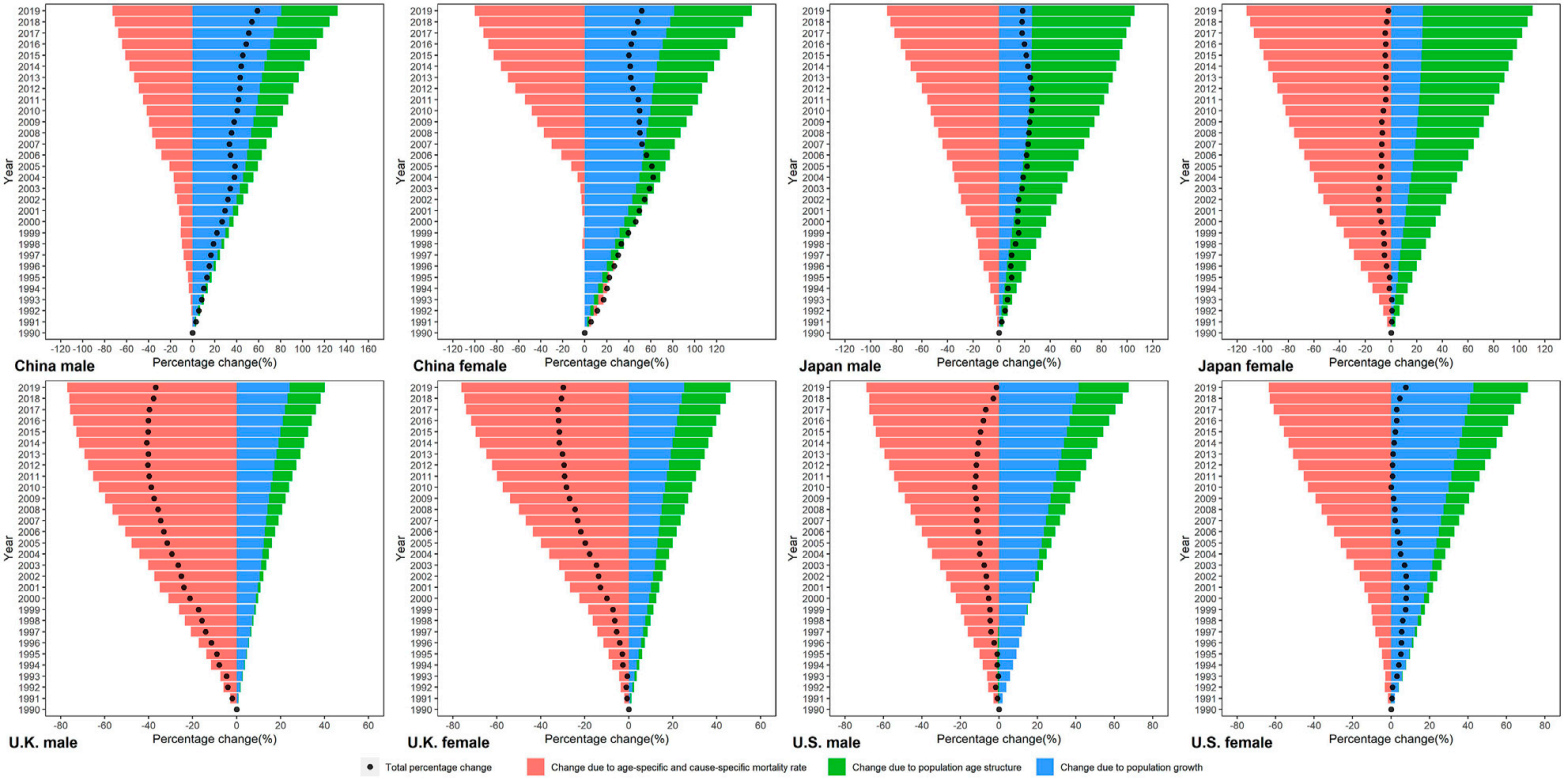
Trends of the age-standardized rates and crude rates per 100,000 population of smoking attributable mortality by sex in China, Japan, United Kingdom, and United States, 1990–2019. Standardized to the GBD 2019 (Global Burden of Disease Study 2019) global age-standard population. (Trends in deaths attributable to smoking in China, Japan, United Kingdom, and United States from 1990 to 2019, Wuhan, China, 2022).

We decomposed the difference in smoking attributable deaths between 1990 and 2019 into three demographic factors (Figure 1). Population growth accounted for increases in numbers of smoking attributable deaths across all four countries, but its contribution ranged from 25.06% in Japanese female to 80.78% in Chinese male. Overall, the contribution of population growth to the increase in smoking attributable deaths was higher in China and US, and lower in Japan and UK. Shifts in population age structures led to increased deaths in all four countries, its relative contribution ranged from 85.44% in Japanese female to 0.24% in UK male. The effect of population age structure changes on smoking-attributable deaths is most pronounced in Japan. Changes in age-specific and cause-specific mortality rate drove reductions in smoking attributable deaths for both sexes in four countries, and its contribution ranged from −112.65% in Japanese female to -63.54% in US female. Except in Chinese male, the relative contributions of changes in age-specific and cause-specific mortality rate were the largest among the three drivers in other population.

### Trends of Smoking Attributable Mortality

**FIGURE 2:**
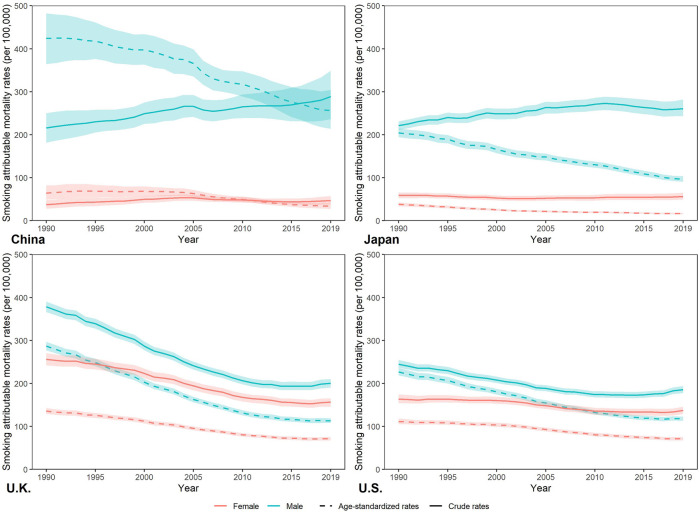
Percentage change in smoking attributable deaths by sex in China, Japan, United Kingdom, and United States in 1990–2019, due to population growth, changes in population age structures, and changes in age-specific and cause-specific mortality. (Trends in deaths attributable to smoking in China, Japan, United Kingdom, and United States from 1990 to 2019, Wuhan, China, 2022).

Figure 2 show the trends of crude, age-standardized smoking attributable mortality in China, Japan, UK, and US from 1990 to 2019. Between 1990 and 2019, smoking attributable crude mortality rate (CMR) increased in Chinese and Japanese male, and decreased in Chinese female, Japanese female. For both sexes in the UK and US, smoking attributable CMR declined from 1990 to 2019. For smoking attributable age-standardized mortality rate (ASMR), it decreased in China, Japan, UK, and US between 1990 and 2019. In 2019, the highest smoking attributable ASMR was for Chinese males (256.65 per 100,000) and the lowest was for Japanese females (16.39 per 100,000). For age-specific smoking attributable mortality (see in Supplementary Figure S3), it increased with age in China, Japan, UK and US Compared to 1990, smoking attributable mortality was reduced in all age groups across countries in 2019.

### APC Analysis for Smoking Attributable Mortality

**FIGURE 3:**
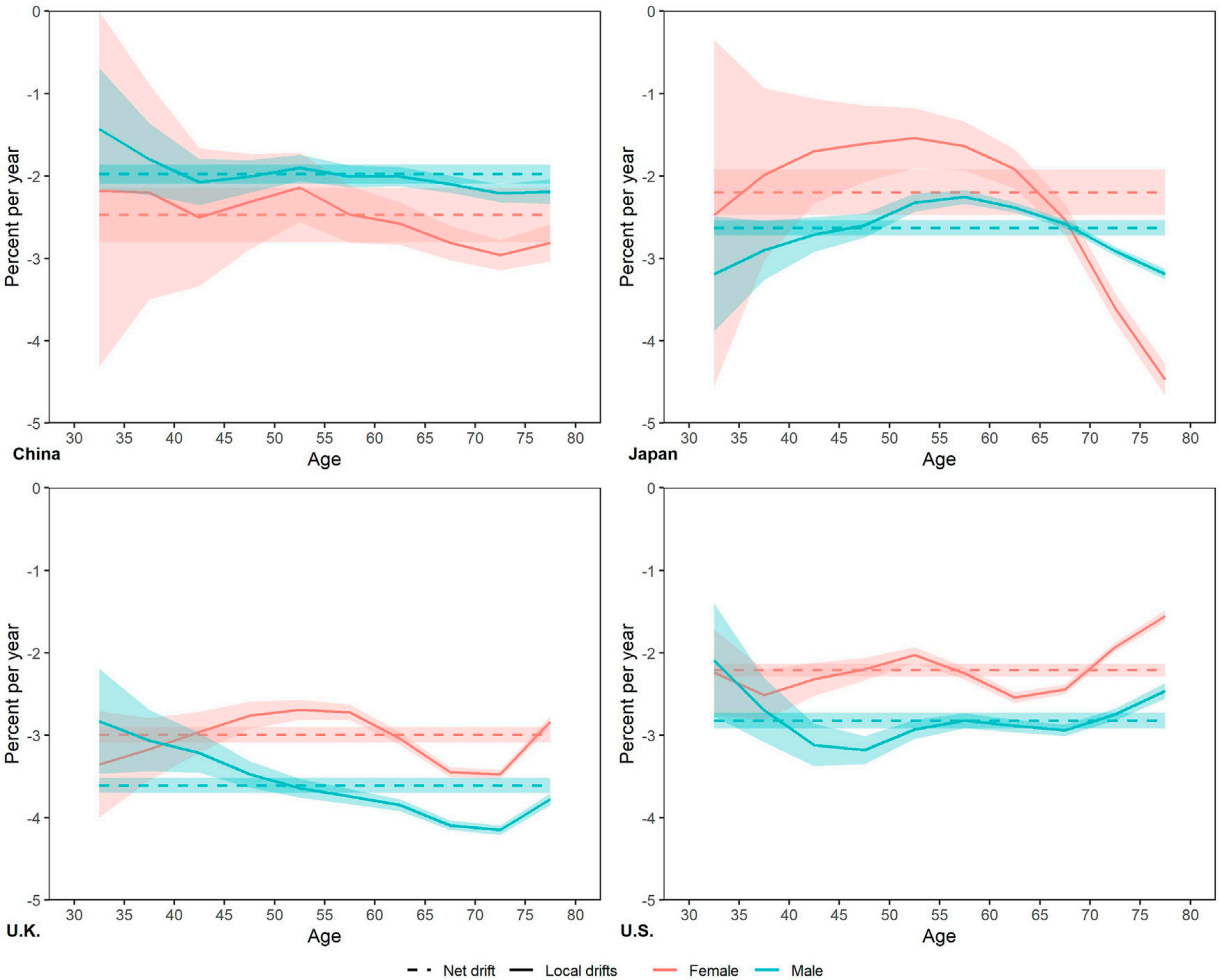
Local drifts with net drift for smoking attributable mortality in China, Japan, United Kingdom, and United States. (Trends in deaths attributable to smoking in China, Japan, United Kingdom, and United States from 1990 to 2019, Wuhan, China, 2022).

From 1990 to 2019, the net drift of smoking attributable mortality was negative in four countries (Figure 3 and Table 1), ranged from Chinese male (−1.98% per year) to UK male (−3.61% per year). The net drift of Chinese males is greater than that of females, while that of Japanese, UK and US females is greater than that of males. The local drifts of each age group in China, UK and US remained relatively stable, close to the net drift. The local drift in Japan is inverted U-shaped with age, and its peak appears at 50–54 age groups in Japan female with −1.54% per year, and peaks in 55–59 age groups in Japan male with −2.25% per year.

**TABLE 1:** Local drifts and net drifts of smoking attributable mortality for males and females in China, Japan, United Kingdom, United States. (Trends in deaths attributable to smoking in China, Japan, United Kingdom, and United States from 1990 to 2019, Wuhan, China, 2022).

	China		Japan		United Kingdom		United States	
	Male	Female	Male	Female	Male	Female	Male	Female
Local drifts								
30–34	−1.43 ± 0.74	−2.18 ± 2.16	−3.19 ± 0.70	−2.47 ± 2.10	−2.83 ± 0.64	−3.35 ± 0.64	−2.09 ± 0.68	−2.24 ± 0.52
35–39	−1.80 ± 0.44	−2.20 ± 1.30	−2.90 ± 0.36	−1.99 ± 1.05	−3.07 ± 0.37	−3.17 ± 0.38	−2.69 ± 0.39	−2.51 ± 0.30
40–44	−2.08 ± 0.28	−2.50 ± 0.84	−2.71 ± 0.21	−1.70 ± 0.63	−3.21 ± 0.24	−2.96 ± 0.25	−3.12 ± 0.26	−2.32 ± 0.20
45–49	−2.01 ± 0.20	−2.31 ± 0.58	−2.60 ± 0.15	−1.61 ± 0.46	−3.47 ± 0.16	−2.76 ± 0.17	−3.18 ± 0.17	−2.20 ± 0.14
50–54	−1.90 ± 0.16	−2.14 ± 0.42	−2.32 ± 0.11	−1.54 ± 0.36	−3.64 ± 0.12	−2.69 ± 0.12	−2.93 ± 0.12	−2.03 ± 0.10
55–59	−2.01 ± 0.14	−2.47 ± 0.35	−2.25 ± 0.08	−1.64 ± 0.29	−3.74 ± 0.09	−2.72 ± 0.10	−2.82 ± 0.09	−2.25 ± 0.08
60–64	−2.01 ± 0.11	−2.58 ± 0.26	−2.38 ± 0.06	−1.91 ± 0.24	−3.85 ± 0.07	−3.04 ± 0.08	−2.89 ± 0.08	−2.54 ± 0.06
65–69	−2.10 ± 0.11	−2.81 ± 0.21	−2.58 ± 0.05	−2.53 ± 0.19	−4.09 ± 0.06	−3.45 ± 0.06	−2.94 ± 0.07	−2.44 ± 0.06
70–74	−2.21 ± 0.11	−2.96 ± 0.19	−2.91 ± 0.05	−3.60 ± 0.17	−4.15 ± 0.06	−3.47 ± 0.05	−2.75 ± 0.07	−1.93 ± 0.05
75–79	−2.19 ± 0.15	−2.81 ± 0.22	−3.19 ± 0.06	−4.47 ± 0.19	−3.78 ± 0.07	−2.84 ± 0.07	−2.46 ± 0.10	−1.55 ± 0.07
Net drift	−1.98 ± 0.12	−2.48 ± 0.33	−2.63 ± 0.09	−2.20 ± 0.28	−3.61 ± 0.09	−3.00 ± 0.09	−2.82 ± 0.10	−2.21 ± 0.08

**FIGURE 4:**
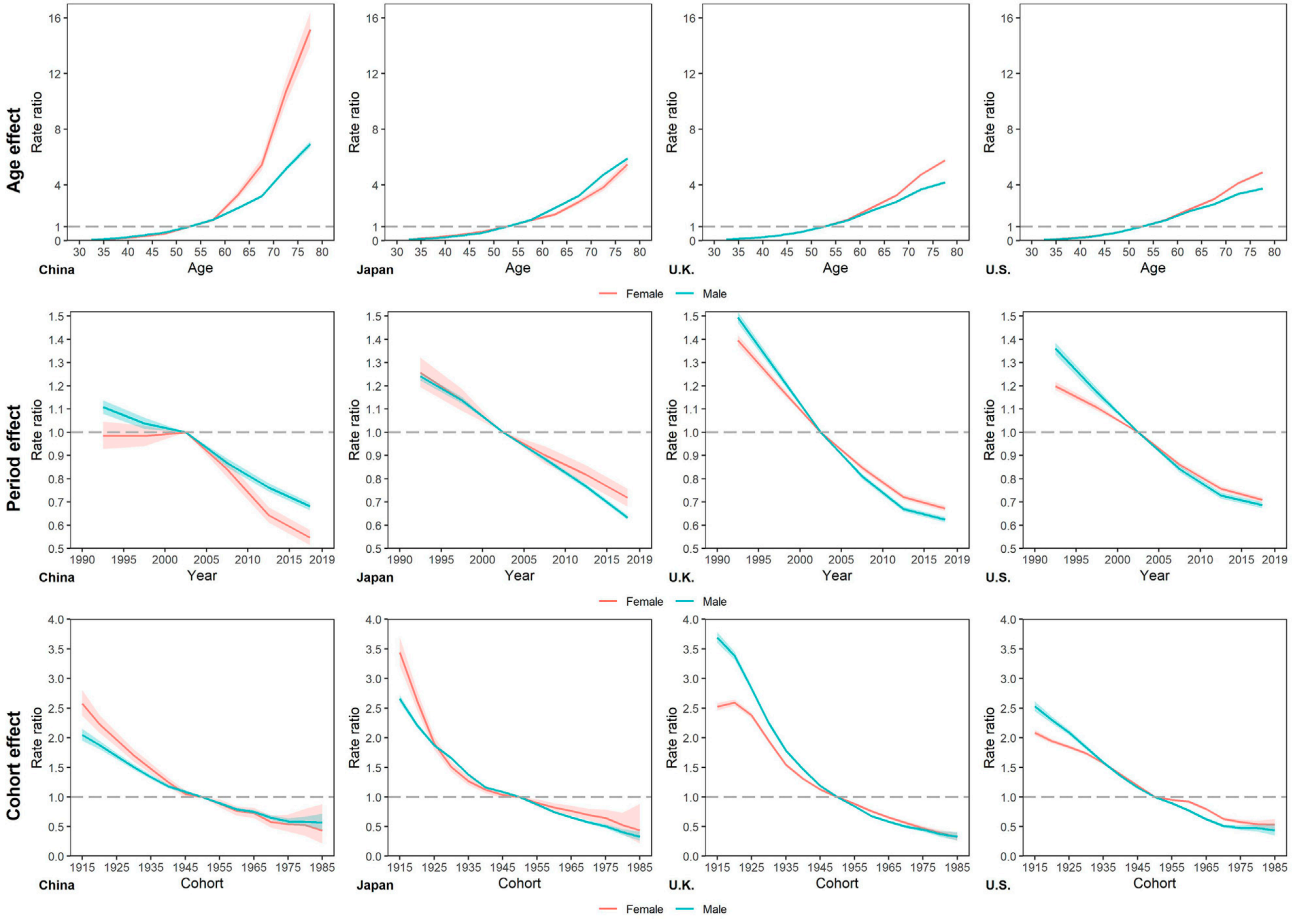
Longitudinal age rate ratios, period rate ratios, and cohort rate ratios of smoking attributable mortality in China, Japan, United Kingdom, and United States. (Trends in deaths attributable to smoking in China, Japan, United Kingdom, and United States from 1990 to 2019, Wuhan, China, 2022).

The longitudinal age RRs of smoking attributable mortality increased with age in all four countries (Figure 4 and Supplementary Table S1), with the largest increase in China. Except for Japan, age RRs of female increased faster than that of male. In the other three countries of this study. The highest age RRs in the 75–79 age group were Chinese female with 15.15, the lowest is 3.73 in US male. Both period RRs and cohort RRs of smoking attributable mortality showed similar monotonic decreasing patterns trend with year in four countries. For period RRs, males declined faster than females in Japan, UK, US, whereas in China the opposite. The percentage change in RRs over the period ranged from UK males (−58.20%, 1.49 to 0.62) to Chinese males (−38.50%, 1.11 to 0.68). And for cohort RRs, the largest decrease was in UK males (from 3.69 to 0.33, a decrease of 91.03%), while the smallest decrease was in Chinese male (from 2.05 to 0.57, a decrease of 72.30%).

Furthermore, as revealed by the results of the Wald test for the APC model (Table 2), the local drifts and net drifts, age, period, and cohort deviations were statistically significant (*p* < 0.01) for both sexes in four countries.

**TABLE 2:** Statistical parameters for overall and age-specific annual percent changes in age–period–cohort models. (Trends in deaths attributable to smoking in China, Japan, United Kingdom, and United States from 1990 to 2019, Wuhan, China, 2022).

	All local drifts = the net drift		Net drift = 0		All period rate ratios = 1		All cohort rate ratios = 1	
	Wald tests	*p*-value	Wald tests	*p*-value	Wald tests	*p*-value	Wald tests	*p*-value
China								
Male	16.87	<0.01	1,088.47	<0.01	1,312.33	<0.01	3,095.05	<0.01
Female	15.5	<0.01	210.98	<0.01	469.04	<0.01	1,488.01	<0.01
Japan								
Male	357.7	<0.01	3,205.88	<0.01	3,603.49	<0.01	20,678.09	<0.01
Female	350.7	<0.01	236.96	<0.01	242.19	<0.01	2,758.07	<0.01
United Kingdom								
Male	249.72	<0.01	5,776.21	<0.01	5,914.88	<0.01	33,206.62	<0.01
Female	627.12	<0.01	3,776.49	<0.01	3,830.3	<0.01	22,990.41	<0.01
United States								
Male	108.88	<0.01	3,133.17	<0.01	3,173.5	<0.01	11,853.83	<0.01
Female	602.69	<0.01	3,116.12	<0.01	3,290.34	<0.01	11,432.05	<0.01

## Discussion

The study found that smoking attributable deaths increased significantly among elderly, especially those over 80, in China, Japan and US from 1990 to 2019. The net drifts of smoking attributable mortality rate were negative in all four countries in this study, with the UK hold the smallest net drift. The local drifts in Japan were inverted U-shaped. The longitudinal age RRs of smoking attributable mortality showed an increasing trend with age, and both period RR and cohort RR for smoking attributable mortality decreased with year. Chinese male had a smaller percentage decrease in period and cohort RR than Chinese female, while this sex differences was reversed in the other three countries.

The UK population had the smallest net drift of smoking attributable mortality rate, i.e., they had the fastest decline in mortality rate between 1990 and 2019. This may be related to the effective tobacco control measures in the UK. Through smoke-free policies [23], provision of help to quit tobacco use [24], and raising the minimum age for purchase of cigarette [25], smoking prevalence in the UK has been effectively controlled and induced a reduction in smoking attributable mortality. The 50–60 age group in Japan had the largest local drifts, that is, they had the slowest declines in mortality rates attributed to smoking, while mortality rates declined more rapidly in the youth and elderly. Smoking cessation among the elderly in Japan may be one of the reasons for this phenomenon [26]. Existing studies have shown that age is an important predictor of smoking cessation rates in Japan [27]. Smoking cessation in older adults may be related to their increased awareness and attention to health risks associated with smoking [28, 29]. In addition, Japan’s promotion of health guidance for people over the age of 40 might also be related to smaller local drifts in elderly [30].

Age has an important effect on smoking attributable mortality rates. With age, smokers and experience more risk factor accumulation over the life course. The leading causes of death from smoking are non-communicable diseases such as lung cancer, IHD and COPD, for which age is an important demographic risk factor [31, 32]. Longitudinal age RRs were higher in the Chinese population, and female were higher than male in the 75–79 age group. The higher prevalence of smoking in China may account for the greater increase in longitudinal age RR. According to the Global Adult Tobacco Survey 2018, the smoking prevalence in China was 26.6% [33]. Due to social customs and cultural reasons, Chinese society is not tolerant of young women smoking. This has resulted in smoking being rare among young Chinese women, but relatively common among older women [34, 35]. The decomposition of changes in smoking attributable deaths in Japan highlights the important impact of population aging. According to the United Nations’ World Aging 2019, the proportion of people over the age of 65 will rise from 9.3% in 2020 to 11.7% in 2030. Considering that 69.95% of smoking-related deaths occur in people over 65 years of age, smoking control deserves more attention in order to achieve the SGDs 3.2 reduce premature mortality from non-communicable diseases by one third through prevention and treatment”.

The decline in period RRs over time may be related to advances in medical technology, and effective control of tobacco use. Previous studies have shown that the measures most associated with reductions in tobacco use maybe increased taxes [36]. The effectiveness of smoke-free policies in reducing smoking is well established [37, 38]. The reinforcement of smoke-free policies has greatly contributed to the reduction in mortality rate attributed to smoking [37, 39]. Better health education and promotion, including pictorial health warnings on tobacco package labelling [24], have led to greater awareness of the harmful effects of smoking, thereby reducing smoking attributable mortality [1]. Improved treatments for smoking-related diseases such as COPD have contributed to decreased smoking attributable mortality [40]. Additionally, urbanization and higher coverage of medical insurance have enabled more people to benefit from improved medical technology [31]. Although the period RR of smoking attributable mortality were decreased between 1990 and 2019 in all four countries, the percentage decrease was the smallest in Chinese males. According to WHO, age-standardized smoking prevalence rate decreased in all four countries from 2000 to 2019 [41], with the smallest decrease among Chinese male (Supplementary Table S2). The age-standardized smoking prevalence rate among Chinese male changed from 50.3% in 2000 to and 45.6% in 2020, respectively. For comparison, age-standardized smoking prevalence rate among Japanese male declined from 52% in 2000 to 30.8% in 2019, and that among male in UK and US declined from 39.2% to 32.7% in 2000 to 17.9% and 22.8% in 2019, respectively. Considering the lag between smoking behavior and smoking related deaths [42, 43], the future of smoking attributable mortality among Chinese male is still not promising and reflects the urgent need for stronger tobacco control interventions.

Decreased cohort RRs were associated with negative societal perceptions of smoking attitudes, improved education, and increased awareness of the health risks of smoking. Previous studies suggest smoking prevalence declined with increasing education attainment [44, 45]. Reinforcement of tobacco control policies could lead to widespread perceptions of smoking denormalization perception [46], which could affect adolescents, the largest group for first smoking experience. And the elevated rate of adolescent smoking in recent years is a cause for concern [42, 47, 48]. Some studies suggests that if youngsters do not smoke in their early adulthood, they are very unlikely to start smoking later in life [49–51]. Considering the above highlights the importance and effectiveness of early prevention and control efforts for youth smoking.

There were gender differences in the net drift, period RRs and cohort RRs of smoking attributable mortality, while China had gender differences in the reverse direction compared to the other three countries. In China, the net drift was larger in males than in females, and the percentage reductions in period and cohort RRs were smaller than in females. And this is the opposite of the other three countries. This inconsistent gender difference may be related to social culture. Chinese males have a much higher smoking prevalence than females, and start smoking at an earlier age than females [52]. Although the Chinese society has been influenced by the western culture in recent decades, it remains quite conservative. The society is quite tolerant to male smoking, but holds a negative view to female smoking [53]. These sociocultural characteristics might account for reversed gender differences in the APC analysis of smoking attributable mortality in China.

This study still has several limitations. Firstly, data on smoking status are self-reported, which might lead to underestimates in countries with low social acceptability of smoking, particularly among females in Asia. Secondly, the scope of this study focuses on smoked tobacco and does not include smokeless tobacco, e-cigarettes, or heated tobacco products. Additionally, this analysis focused on the health effects of primary smoking, excluding the additional harm caused by secondhand smoke. Thirdly, similar to other APC analyses, ecological fallacies can arise because interpretations of findings at the population level may not apply at the individual level. Therefore, further studies are needed to confirm findings based on individual-level studies.

In conclusion, more attention needs to be paid to increased smoking attributable deaths among elderly. Smoke-free policies, provision of help to quit tobacco use, increased taxes might be associated to the decreased age-standardized smoking attributable mortality rate in China, Japan, UK, and US Improved health education and promotion, better access to health care services, and advances in the treatment of smoking-related diseases have also contributed to the decline in smoking attributable mortality. The reversed gender differences in net drift and period RRs, cohort RRs between China and the other three countries may be related to different sociocultural attitudes toward smoking in both sexes.

## Data Availability

The datasets analysed during the current study are available in the GBD Results Tool repository, https://ghdx.healthdata.org/gbd-results-tool.

## Supplementary Material

Supplementary Table S1 The rate ratios of smoking attributable mortality due to age, period, and cohort effects in China, Japan, United Kingdom, and United States (Wuhan, China, 2022).

Supplementary Table S2 Smoking prevalence in China, Japan, United Kingdom, United States in 2000 and 2019 (Wuhan, China, 2022).
