# Supplementary material for: Trends in Deaths Attributable to Smoking in China, Japan, United Kingdom, and United States From 1990 to 2019
Source: Int J Public Health. 2022 Sep 15;67:1605147. doi: 10.3389/ijph.2022.1605147 (PMC9519860; doi:10.3389/ijph.2022.1605147)
Supplement: Supplementary file 1 [file DataSheet1.ZIP › revised supplementary file/revised-Supplementary file.docx]

**Journal: International journal of public health**

**Manuscript name: Trends in deaths attributable to smoking in China, Japan, the United Kingdom, and the United States from 1990 to 2019**

**Table S1** The rate ratios of smoking attributable mortality due to age, period, and cohort effects in China, Japan, the United Kingdom, and the United States.

| Factor | China | |  | Japan | |  | The United Kingdom | |  | The United States | |
| --- | --- | --- | --- | --- | --- | --- | --- | --- | --- | --- | --- |
|  | male | female |  | male | female |  | male | female |  | male | female |
| Age |  |  |  |  |  |  |  |  |  |  |  |
| 30-34 | 0.07±0.01 | 0.08±0.02 |  | 0.09±0.01 | 0.09±0.02 |  | 0.09±0.01 | 0.11±0.01 |  | 0.06±0.01 | 0.07±0.01 |
| 35-39 | 0.17±0.01 | 0.14±0.03 |  | 0.17±0.01 | 0.23±0.03 |  | 0.18±0.01 | 0.19±0.01 |  | 0.14±0.01 | 0.15±0.01 |
| 40-44 | 0.36±0.02 | 0.29±0.04 |  | 0.33±0.01 | 0.40±0.04 |  | 0.34±0.01 | 0.35±0.01 |  | 0.29±0.01 | 0.31±0.01 |
| 45-49 | 0.59±0.02 | 0.49±0.05 |  | 0.57±0.01 | 0.65±0.05 |  | 0.60±0.02 | 0.60±0.02 |  | 0.58±0.02 | 0.58±0.01 |
| 50-54 | 1.00 | 1.00 |  | 1.00 | 1.00 |  | 1.00 | 1.00 |  | 1.00 | 1.00 |
| 55-59 | 1.50±0.05 | 1.49±0.12 |  | 1.49±0.03 | 1.48±0.10 |  | 1.46±0.03 | 1.50±0.03 |  | 1.47±0.03 | 1.48±0.03 |
| 60-64 | 2.32±0.07 | 3.23±0.25 |  | 2.35±0.04 | 1.89±0.12 |  | 2.18±0.04 | 2.39±0.05 |  | 2.14±0.05 | 2.27±0.04 |
| 65-69 | 3.20±0.10 | 5.45±0.43 |  | 3.22±0.06 | 2.78±0.18 |  | 2.79±0.06 | 3.26±0.07 |  | 2.62±0.06 | 2.99±0.05 |
| 70-74 | 5.15±0.17 | 10.72±0.85 |  | 4.73±0.08 | 3.82±0.24 |  | 3.68±0.08 | 4.74±0.10 |  | 3.36±0.08 | 4.13±0.08 |
| 75-79 | 6.93±0.23 | 15.15±1.21 |  | 5.90±0.11 | 5.47±0.35 |  | 4.19±0.09 | 5.77±0.13 |  | 3.73±0.09 | 4.91±0.09 |
| Period |  |  |  |  |  |  |  |  |  |  |  |
| 1990-1994 | 1.11±0.03 | 0.99±0.06 |  | 1.24±0.02 | 1.26±0.06 |  | 1.49±0.02 | 1.40±0.02 |  | 1.36±0.03 | 1.20±0.02 |
| 1995-1999 | 1.04±0.02 | 0.98±0.04 |  | 1.14±0.01 | 1.14±0.05 |  | 1.25±0.02 | 1.19±0.02 |  | 1.17±0.02 | 1.11±0.01 |
| 2000-2004 | 1.00 | 1.00 |  | 1.00 | 1.00 |  | 1.00 | 1.00 |  | 1.00 | 1.00 |
| 2005-2009 | 0.87±0.02 | 0.84±0.04 |  | 0.89±0.01 | 0.90±0.04 |  | 0.81±0.01 | 0.85±0.01 |  | 0.84±0.01 | 0.86±0.01 |
| 2010-2014 | 0.76±0.02 | 0.64±0.03 |  | 0.77±0.01 | 0.82±0.04 |  | 0.67±0.01 | 0.72±0.01 |  | 0.73±0.01 | 0.76±0.01 |
| 2015-2019 | 0.68±0.02 | 0.55±0.03 |  | 0.63±0.01 | 0.72±0.04 |  | 0.62±0.01 | 0.67±0.01 |  | 0.69±0.01 | 0.71±0.01 |
| Cohort |  |  |  |  |  |  |  |  |  |  |  |
| 1915 | 2.05±0.10 | 2.58±0.22 |  | 2.66±0.06 | 3.44±0.24 |  | 3.69±0.09 | 2.53±0.06 |  | 2.53±0.08 | 2.09±0.05 |
| 1920 | 1.88±0.07 | 2.23±0.15 |  | 2.21±0.04 | 2.63±0.16 |  | 3.38±0.07 | 2.59±0.05 |  | 2.30±0.06 | 1.94±0.04 |
| 1925 | 1.69±0.05 | 1.97±0.12 |  | 1.87±0.03 | 1.91±0.11 |  | 2.83±0.05 | 2.38±0.04 |  | 2.09±0.04 | 1.85±0.03 |
| 1930 | 1.51.00.04 | 1.70±0.10 |  | 1.66±0.02 | 1.51±0.08 |  | 2.25±0.04 | 1.95±0.04 |  | 1.84±0.04 | 1.74±0.03 |
| 1935 | 1.34±0.04 | 1.48±0.09 |  | 1.38±0.02 | 1.27±0.07 |  | 1.79±0.03 | 1.54±0.03 |  | 1.59±0.03 | 1.58±0.03 |
| 1940 | 1.18±0.03 | 1.25±0.07 |  | 1.16±0.02 | 1.13±0.06 |  | 1.47±0.03 | 1.31±0.02 |  | 1.36±0.03 | 1.38±0.02 |
| 1945 | 1.09±0.03 | 1.05±0.06 |  | 1.09±0.02 | 1.03±0.06 |  | 1.19±0.02 | 1.13±0.02 |  | 1.16±0.02 | 1.19±0.02 |
| 1950 | 1.00 | 1.00 |  | 1.00 | 1.00 |  | 1.00 | 1.00 |  | 1.00 | 1.00 |
| 1955 | 0.90±0.03 | 0.89±0.07 |  | 0.88±0.02 | 0.90±0.06 |  | 0.84±0.02 | 0.89±0.02 |  | 0.90±0.02 | 0.95±0.02 |
| 1960 | 0.79±0.03 | 0.76±0.08 |  | 0.75±0.02 | 0.82±0.07 |  | 0.68±0.02 | 0.76±0.02 |  | 0.77±0.02 | 0.92±0.02 |
| 1965 | 0.75±0.03 | 0.73±0.09 |  | 0.66±0.02 | 0.77±0.08 |  | 0.58±0.02 | 0.65±0.02 |  | 0.63±0.02 | 0.80±0.02 |
| 1970 | 0.65±0.04 | 0.58±0.10 |  | 0.57±0.03 | 0.70±0.10 |  | 0.50±0.03 | 0.56±0.03 |  | 0.51±0.03 | 0.63±0.03 |
| 1975 | 0.59±0.05 | 0.54±0.14 |  | 0.51±0.03 | 0.65±0.13 |  | 0.45±0.03 | 0.47±0.04 |  | 0.48±0.04 | 0.58±0.04 |
| 1980 | 0.58±0.08 | 0.53±0.23 |  | 0.41±0.05 | 0.52±0.18 |  | 0.37±0.05 | 0.39±0.05 |  | 0.48±0.06 | 0.54±0.05 |
| 1985 | 0.57±0.14 | 0.43±0.33 |  | 0.33±0.08 | 0.44±0.33 |  | 0.33±0.07 | 0.32±0.07 |  | 0.44±0.10 | 0.53±0.09 |

**Table S2** Smoking prevalence in China, Japan, the United Kingdom, the United States in 2000 and 2019

|  |  | China | Japan | The United Kingdom | The United States |
| --- | --- | --- | --- | --- | --- |
| 2000 | Male | 50.3 | 52.0 | 39.2 | 32.7 |
|  | Female | 3.2 | 14.5 | 36.5 | 24.4 |
| 2019 | Male | 45.6 | 30.8 | 17.9 | 22.8 |
|  | Female | 1.7 | 10.2 | 14.2 | 16.3 |

**Figure S1** The smoking attributable deaths and percentage changes in China, Japan, the United Kingdom, the United States, 1990 and 2019. (Wuhan, China, 2022)

**Figure S2** The smoking attributable deaths by age group in China, Japan, the United Kingdom, the United States, 1990 and 2019. (Wuhan, China, 2022)

**Figure S3** Age-specific smoking attributable mortality rates in China, Japan, the United Kingdom, and the United States in 1990 and 2019. (Wuhan, China, 2022)
