# Supplementary figures and images for: Trends in Deaths Attributable to Smoking in China, Japan, United Kingdom, and United States From 1990 to 2019
Source: Int J Public Health. 2022 Sep 15;67:1605147. doi: 10.3389/ijph.2022.1605147 (PMC9519860; doi:10.3389/ijph.2022.1605147)

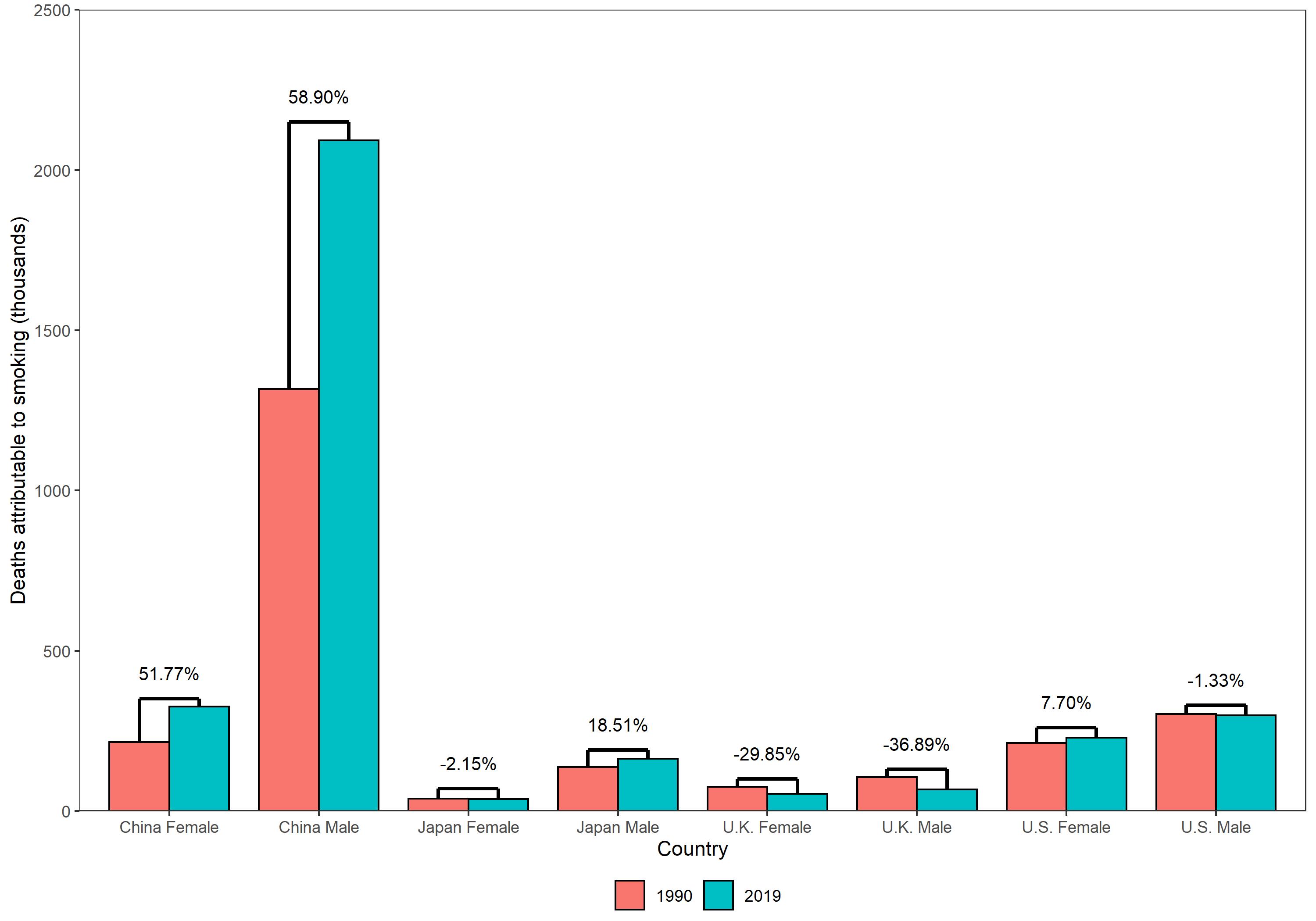

Supplement: Supplementary file 1 [file DataSheet1.ZIP › revised supplementary file/sfigure 1.jpeg]

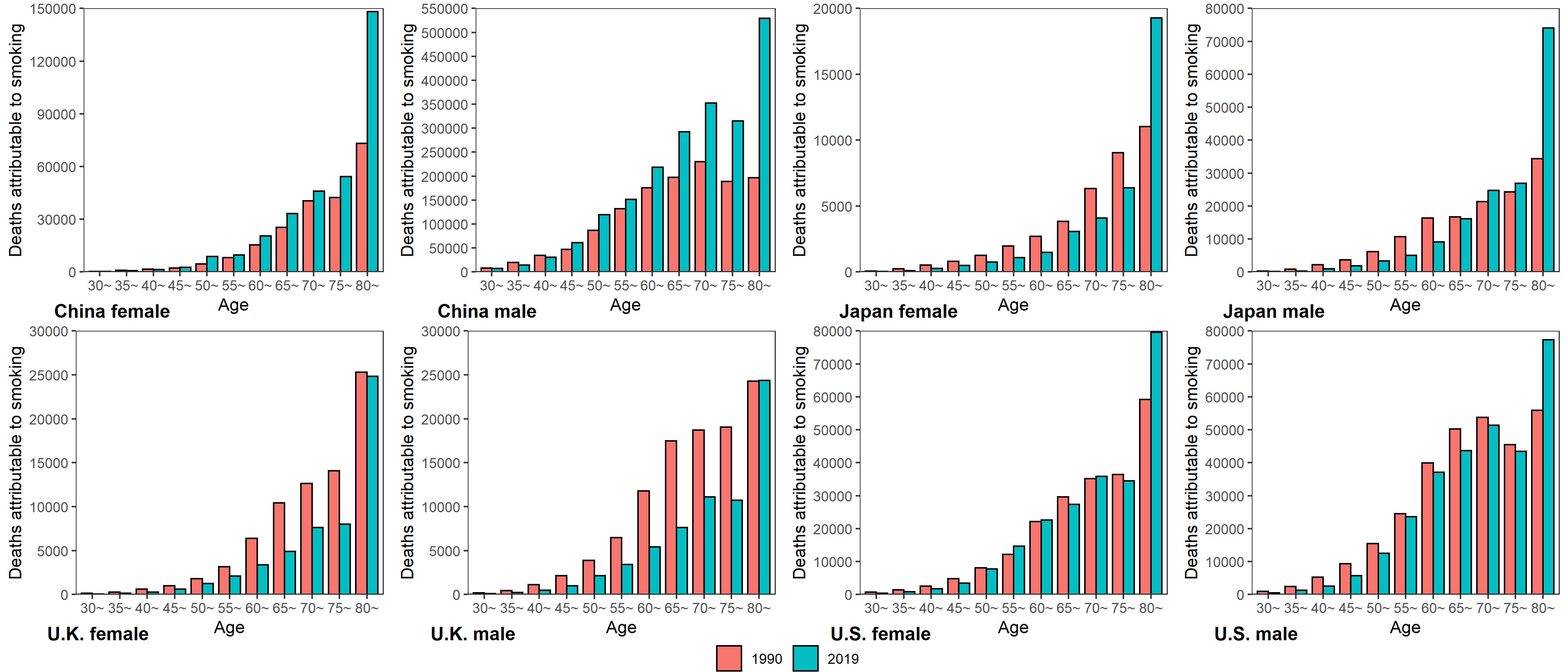

Supplement: Supplementary file 1 [file DataSheet1.ZIP › revised supplementary file/sfigure 2.jpeg]

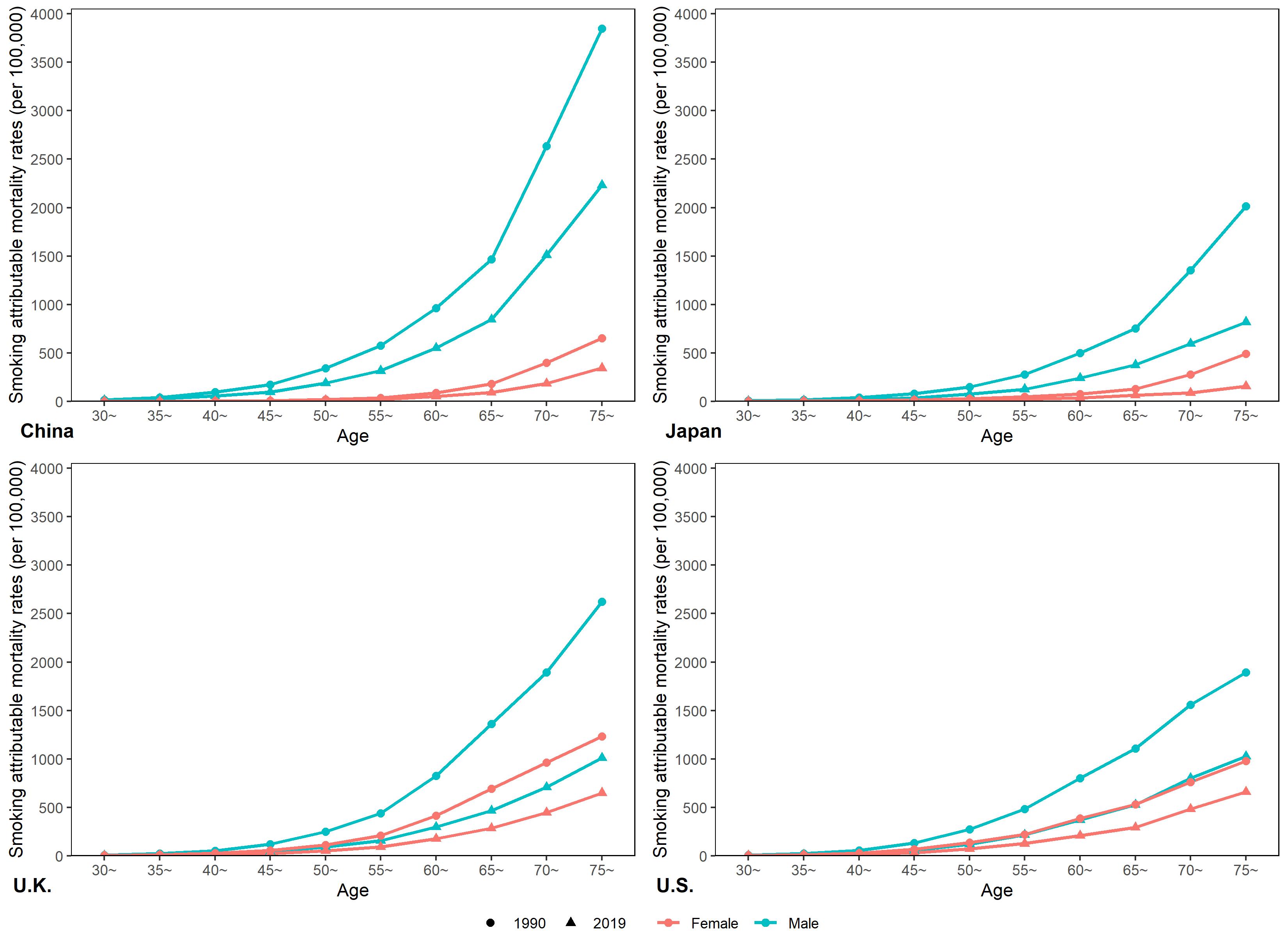

Supplement: Supplementary file 1 [file DataSheet1.ZIP › revised supplementary file/sfigure 3.jpeg]
